# Consumption of isoflurane and recovery time in low-flow anaesthesia versus high-flow anaesthesia

**DOI:** 10.12669/pjms.42.9.15941

**Published:** 2026-09

**Authors:** Aaisha Hanif, Syed Asadullah Jafri, Amjad Nadeem, Maryyam Hanif

**Affiliations:** 1 Aaisha Hanif, MBBS. Completed Anaesthesiology Residency, CMH Malir Cantt, Karachi and KRL Hospital Islamabad, Pakistan; 2 Syed Asadullah Jafri, FCPS Anaesthesiology Consultant Anesthesia, Combined Military Hospital (CMH), Lahore, Pakistan; 3 Amjad Nadeem, FCPS Anaesthesiology Assistant Professor Anesthesia KRL Hospital, Islamabad, Pakistan; 4 Maryyam Hanif, FCPS Anaesthesiology Locum Consultant Anesthesia Aga Khan University Hospital (AKUH), Karachi, Pakistan

**Keywords:** Anesthesia, Flow, High, Inhalational, Isoflurane, Low, Outcome

## Abstract

**Objective:**

To compare the effects of low-flow versus high-flow Isoflurane anesthesia on volatile anesthetic consumption, recovery characteristics, postoperative inflammatory response, and patient clinical outcomes in elective laparoscopic cholecystectomy.

**Methodology:**

This quasi-experimental study was conducted at Department of Anesthesia, Combined Military Hospital, Malir Cantt, Karachi, Pakistan from October 2022-March 2023. A total of 280 consecutive eligible patients fulfilling the inclusion criteria were recruited using non-probability convenience sampling and assigned into Group-A (Low-flow anesthesia) (n=140) and Group-B (High-flow anesthesia) (n=140). Primary variables were the mean intraoperative isoflurane consumption, and the neutrophil-to-lymphocyte ratio (NLR) at 24 hours postoperatively. Secondary variables were patient outcomes and anesthesia adverse effect profile.

**Results:**

Mean isoflurane consumption was significantly lower in Group-A at 12.04 ± 3.00 mL, compared with 16.18 ± 3.79 mL in Group-B (p<0.001). Median baseline NLR values were nearly the same: 2.27 (0.87) in Group-A and 2.30 (0.89) in Group-B (p=0.972). However, postoperative NLR at 24 hours showed a clear rise in Group-B, recorded as 4.46 (2.01), compared with 3.45 (1.54) in Group-A (p<0.001). Low-flow anesthesia was associated with shorter time to extubation while time to Aldrete score ≥ 9, postoperative pain score, rescue analgesia requirements, postoperative nausea and vomiting, shivering, hypothermia, delayed emergence, dizziness and HDU admissions were comparable between groups.

**Conclusion:**

The study supports low flow anaesthesia as an effective technique that reduces anesthetic use, shortens the extubation time and attenuated the postoperative inflammatory response without increasing perioperative adverse events related to anesthesia or delaying recovery.

## INTRODUCTION

General anesthesia remains the preferred mode for major surgical procedures worldwide despite newer options of regional anesthesia. Patient outcomes following general anesthesia remain a critical domain of research, especially as anesthetic practices evolve to optimize safety, cost-effectiveness, and environmental impact. Among these practices, the use of low-flow anesthesia (LFA), defined as reduced fresh gas flow (FGF) rates during maintenance, has gained traction over the past decade.^1^ The theoretical advantages of LFA include decreased consumption of volatile agents and oxygen,^2^ hence the cost,^3^ improved conservation of heat and humidity in inspired gases,^4^ and reduced ecological footprint.^5^,^6^ However, there exists limited and conflicting evidence regarding whether LFA alters patient clinical outcomes and recovery especially when using commonly utilized agents such as Isoflurane compared to high-flow or standard-flow (high-flow) anesthesia.

While most contemporary studies investigating low-flow versus high-flow techniques have employed agents like Sevoflurane or Desflurane, extrapolation of these findings to isoflurane remains uncertain due to differences in pharmacokinetics, solubility, and metabolism among volatile agents. Comparison studies have demonstrated that low-flow regimes preserve mucociliary function and respiratory mechanics better than high-flow techniques when using desflurane or nitrous oxide–based systems.^7^ Similarly, pulmonary function studies following laparoscopic surgery under sevoflurane-based low-flow and high-flow anesthesia protocols found no significant postoperative decline attributable to the flow rate.^8^ Available research suggests that low-flow anesthesia may attenuate inflammatory,^9^,^10^ and oxidative stress responses associated with surgery.^11^ while maintaining adequate tissue oxygenation.^12^

Given that laparoscopic cholecystectomy is a widely performed procedure with relatively consistent operative time, standardized pneumoperitoneum, and minimal tissue trauma compared to open surgery, it presents an ideal clinical model to examine the isolated influence of anesthetic flow rate on postoperative outcomes. Our goal is to provide robust, contemporary evidence to inform anesthetic protocols, optimize recovery, minimize perioperative inflammation, and potentially reduce costs and environmental impact without compromising patient safety or physiological stability.

## METHODOLOGY

This quasi-experimental study was carried out at the Department of Anesthesia, Combined Military Hospital, Malir Cantt, Karachi, Pakistan from October 2022- March 2023. The sample size for two groups was calculated keeping the confidence interval at 95%, power of test at 80%, with the anticipated mean difference between inhalational anesthesia consumption between low flow versus high flow anesthesia being 17.90±4.18 ml versus 28.96±2.34 ml.^13^ Comparing the mean difference between both groups of 11.06±1.84 ml, the minimum sample size came out to be 129 patients for each group. A total of 280 consecutive eligible patients fulfilling the inclusion criteria who presented for elective laparoscopic cholecystectomy were recruited using non-probability convenience sampling during the study period. (Fig.1).

**Fig-1:**
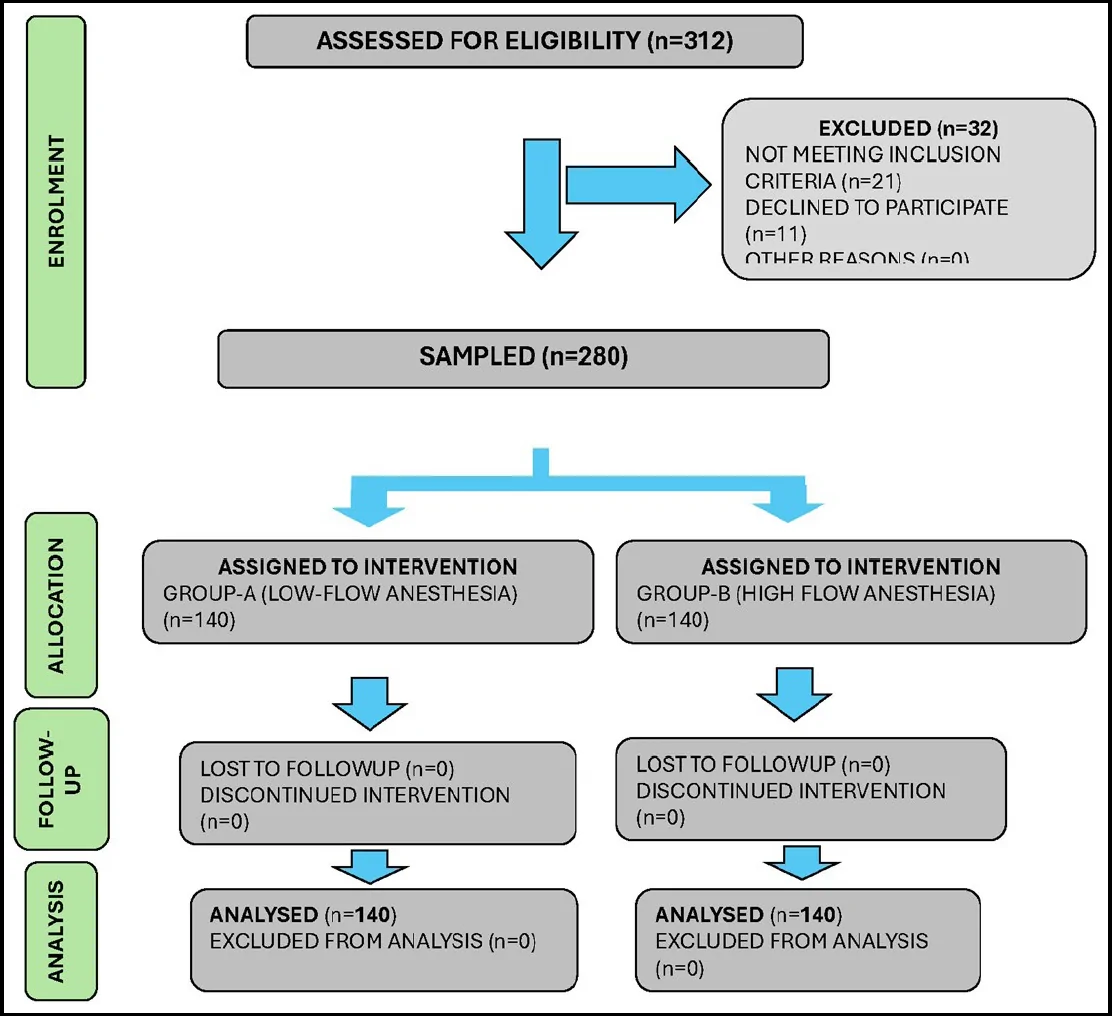
Patient flow diagram.

### Ethical Approval:

It was obtained from the ethical review board vide letter no: 89/2022/Trg/ERC, dated: September 22, 2022

### Inclusion criteria:

Included patients of both genders, ages 18-65 years scheduled for an elective laparoscopic cholecystectomy under general anesthesia by an endotracheal tube

### Exclusion criteria:

Comprised of patients with significant pre-existing respiratory disease, clinically important ischemic heart disease or valvular lesions, uncontrolled hypertension, decompensated liver or renal dysfunction, known hematologic or systemic inflammatory disease, active infection, or malignancy, morbid obesity with body mass index greater than 35 kg/m², pregnancy or lactation, known or suspected allergy to any study medication, chronic use of systemic corticosteroids or other immunosuppressive drugs, history of difficult airway or anticipated difficult intubation, psychiatric illness that might affect consent or postoperative assessment, and refusal or inability to provide informed consent and cases converted intraoperatively to open cholecystectomy or with major protocol deviations including change of volatile agent, and prolonged pneumoperitoneum beyond two hours. The study method included all patients as per the inclusion criteria furnished. The patients were assigned into two groups of 140 patients each. Group A (n=140) to receive low-flow isoflurane anesthesia and Group B (n=140) to receive high-flow isoflurane anesthesia.

All potentially eligible patients were identified from the outpatient department (OPD) surgical clinics where they were evaluated and scheduled for elective laparoscopic cholecystectomy. These patients were approached consecutively and recruited using non-probability convenience sampling. On the preoperative visit, the study was explained in detail in the patient’s preferred language. Written informed consent was obtained. Participation was entirely voluntary and patients were assured that refusal would not affect their standard of care. The study protocol was reviewed and approved by the Institutional Ethics Review Committee, and it adhered to the ethical principles of the Declaration of Helsinki.

Participants were assigned consecutively to Group-A and Group-B according to the study protocol: Group-A (n = 140), receiving low-flow isoflurane anesthesia, and Group-B (n = 140), receiving high-flow isoflurane anesthesia.

In both groups, anesthesia was induced with a standardized regimen comprising Intravenous Propofol at 1.5 mg/kg, IV Nalbuphine 0.15 mg/kg, IV Atracurium 0.5 mg/kg after pre-medication with IV Metoclopramide 10 mg and IV Dexamethasone 4 mg. After induction, an initial wash-in period was performed using a fresh gas flow of 4 L/min with an oxygen–air mixture to achieve an inspired oxygen fraction of 0.5 and to allow adequate denitrogenating and equilibration of the circuit. In Group-A (n=140), after the first 10 minutes of anesthesia, fresh gas flow was reduced to 1 L/min, with the oxygen–air ratio adjusted to maintain an inspired oxygen fraction of at least 0.4. In Group-B (n=140), fresh gas flow was maintained at 4 L/min throughout the procedure.^14^ In both groups, isoflurane concentration was titrated to maintain approximately 1.0–1.2 minimum alveolar concentration (MAC),^15^ or with bispectral index (BIS) monitoring value between 40-60.^16^ Ventilation parameters, intraoperative analgesia, antiemetic prophylaxis, and neuromuscular reversal were standardized for all patients to reduce confounding.

Hemodynamic variables, end-tidal carbon dioxide, end-tidal isoflurane concentration, airway pressures, and intraoperative complications were recorded every 15 minutes till extubation.

At the end of surgery, volatile anesthetic agent was switched off and fresh gas flow increased to 6L after giving reversal agent. Postoperative assessments included recovery profile, frequency of anesthesia adverse effects including hypotension, bradycardia, nausea, vomiting as per the study outcomes for the next 12 hours in all patients. Inflammatory markers were assessed at 24 hours in both groups.

Primary variables were the mean intraoperative isoflurane consumption, measured in milliliters from induction to the end of surgery, and the neutrophil-to-lymphocyte ratio (NLR) at 24 hours postoperatively, derived from venous blood samples to assess the postoperative inflammatory response at 24 hours postoperatively.^17^ NLR was selected because of its cost effectiveness. Most important secondary variable was the mean extubation time and was calculated from switching off the Isoflurane till extubation. Secondary variables also included: time required to achieve an Aldrete score of ≥9,^18^ postoperative pain scores at 1, 6, and 24 hours, and the need for rescue analgesia within the first 12 hours postoperatively. Adverse effects related to anesthesia were recorded both intraoperatively and postoperatively, including episodes of hypotension, bradycardia, as well as postoperative nausea or vomiting, shivering, hypothermia, dizziness, delayed emergence, and any unplanned admission to the intensive care unit.

Variables including age, BMI, duration of surgery, duration of pneumoperitoneum, time to extubation, time to Aldrete score, opioid use and hospital stay was expressed as mean±SD and compared using the independent samples t-test. Gender, ASA status, need for rescue analgesia, adverse effects including hypotension, bradycardia, shivering, delayed emergence, hypoxia and hypercarbia were expressed as frequency with percentage and compared using the Chi-square and Fisher-exact test as appropriate.

### Statistical Analysis:

NLR ratio and pain scores were expressed as median with interquartile ranges and compared using the Mann-Whitney U test. Data normality was assessed and data was normally distributed. A *p* value of ≤0.05 was considered statistically significant. All statistical calculations were performed using Statistical Package for Social Sciences 26.0.

## RESULTS

A total of 280 patients were included in the study and assigned into Group-A (Low-flow anesthesia) (n=140) and Group-B (High-flow anesthesia) (n=140). The mean age in Group-A was 43.98 ± 11.13 years, while Group-B showed 46.39 ± 11.40 years (p=0.075). Mean BMI was similar between the groups, recorded as 26.86 ± 3.90 kg/m² in Group-A and 26.85 ± 4.13 kg/m² in Group-B (p=0.986). Gender distribution revealed 71.4% males and 28.6% females in Group-A, compared with 74.3% males and 25.7% females in Group-B (p=0.591). ASA classification was also comparable, with Group-A showing 39.3% ASA-I, 40.0% ASA-II, and 20.7% ASA-III, while Group-B had 33.6% ASA-I, 48.6% ASA-II, and 17.9% ASA-III (p=0.353) (Table-I).

**Table-I:** Demographic and clinical characteristics (n=280).

Variable	Group-A (n=140)	Group-B (n=140)	p value
Mean Age (Years)	43.98±11.13	46.39±11.40	0.075
Mean BMI (KG/M^2^)	26.86±3.90	26.85±4.13	0.986
Gender			
Male	100 (71.4%)	104 (74.3%)	0.591
Female	40 (28.6%)	36 (25.7%)	
ASA Status			
ASA-I	55 (39.3%)	47 (33.6%)	0.353
ASA-II	56 (40.0%)	68 (48.6%)	
ASA-III	29 (20.7%)	25 (17.9%)	

*BMI=Body Mass Index, *ASA= American Society of Anesthesiology Status.

The mean duration of surgery was 60.82 ± 10.00 minutes in Group-A and 61.85 ± 10.41 minutes in Group-B (p=0.401). Mean pneumoperitoneum time was 50.85 ± 10.50 minutes in Group-A and 51.24 ± 10.62 minutes in Group-B (p=0.762). A notable difference observed in mean isoflurane consumption, which was significantly lower in Group-A at 12.04 ± 3.00 mL, compared with 16.18 ± 3.79 mL in Group-B (p<0.001).

Recovery parameters showed slightly faster extubation in Group-A, with a mean time of 8.02 ± 1.87 minutes, compared with 8.59 ± 2.04 minutes in Group-B (p=0.008). The mean time to reach an Aldrete score above 9 was 20.29 ± 4.50 minutes in Group-A and 20.91 ± 5.20 minutes in Group-B (p=0.143). Opioid requirements were similar, recorded as 11.86 ± 4.35 mg in Group-A and 12.64 ± 4.22 mg in Group-B (p=0.063). Mean hospital stay was 2.03 ± 0.58 days versus 2.13 ± 0.58 days (p=0.077). Inflammatory markers demonstrated significant differences at 24 hours. Median baseline NLR values were nearly the same: 2.27 (0.87) in Group-A and 2.30 (0.89) in Group-B (p=0.972). However, postoperative NLR at 24 hours showed a clear rise in Group-B, recorded as 4.46 (2.01), compared with 3.45 (1.54) in Group-A (p<0.001). Pain scores remained comparable: at 1 hour the values were 4.35 ± 2.1 versus 4.60 ± 2.0 (p=0.180), at 6 hours 3.80 ± 2.3 versus 3.50 ± 1.6 (p=0.387), and at 24 hours 2.05 ± 1.7 versus 2.20 ± 1.2 (p=0.385). Rescue analgesia was required in 61 patients (43.6%) in Group-A and 46 patients (32.9%) in Group-B (p=0.065) (Table-II).

**Table-II:** Comparison of operative and postoperative outcomes (n=280).

Variable	Group-A (n=140)	Group-B (n=140)	p value
Mean Duration of Surgery (Min)	60.82±10.00	61.85±10.41	0.401
Mean Duration of Pneumoperitoneum (Min)	50.85±10.50	51.24±10.62	0.762
Mean Isoflurane Consumption (ML)	12.04±3.00	16.18±3.79	<0.001
Mean Time to Extubation (Min)	8.02±1.87	8.59±2.04	0.008
Mean Time to Aldrete Score >9 (Min)	20.29±4.50	20.91±5.20	0.143
Mean Opioid Use (MG/24 HR)	11.86±4.35	12.64±4.22	0.063
Mean Length of Stay (DAYS)	2.03±0.58	2.13±0.58	0.077
Median NLR Value Pre-op	2.27 (0.87)	2.30 (0.89)	0.972
Median NLR Value Post-op at 24 HR	3.45 (1.54)	4.46 (2.01)	<0.001
***Median VAS Post-op Pain Scores***			
At 01 Hours	4.35 (2.1)	4.60 (2.0)	0.180
At 06 Hours	3.80 (2.3)	3.50 (1.6)	0.387
At 24 Hours	2.05 (1.7)	2.20 (1.2)	0.385
Rescue Analgesia Required	61 (43.6%)	46 (32.9%)	0.065

*VAS= Visual Analog Scale, *NLR= Neutrophil to Lymphocyte Ratio.

Assessment of anesthesia related adverse effects showed no significant differences in most variables. Intraoperative hypotension occurred in 29 patients (20.7%) in Group-A and 30 patients (21.4%) in Group-B (p=0.883). Bradycardia occurred in eight patients (5.7%) versus two patients (1.4%) (p=0.636). Intraoperative hypoxia was significantly more frequent in Group-A at 12 patients (8.6%), compared with two patients (1.4%) in Group-B (p=0.011). SpO2 levels in these patients remained between 92-94%. It was corrected by increasing and then readjusting the FiO2. Severe hypoxia requiring protocol deviation did not occur in any patient. Hypercarbia was more common in Group-A at 12 patients (8.6%) versus 3 patients (2.1%) (p=0.030). It was managed by adjusting ventilatory parameters. In one patient, it was attributed to exhaustion of CO2 absorbent. Postoperative nausea and vomiting occurred in 35 patients (25.0%) in Group-A and 47 patients (33.6%) in Group-B (p=0.148). Shivering occurred in 24 patients (17.1%) versus 28 patients (20.0%) (p=0.539), while postoperative hypothermia occurred in 20 patients (14.3%) versus 31 patients (22.1%) (p=0.089). Delayed emergence was seen in 13 patients (9.3%) in Group-A and 8 patients (5.7%) in Group-B (p=0.257). Dizziness occurred in 17 patients (12.1%) versus 15 patients (10.7%) (p=0.707). The requirement for HDU admission was identical in both groups at 5 patients (3.6%) each (p=1.000) (Table-III).

**Table-III:** Anesthsia related adverse effect profile between both groups (n=280).

Variable	Group-A (n=140)	Group-B (n=140)	p value
Intra-op Hypotension	29 (20.7%)	30 (21.4%)	0.883
Intra-op Bradycardia	08 (5.7%)	02 (1.4%)	0.636
Intra-op Hypoxia	12 (8.6%)	02 (1.4%)	0.011
Intra-op Hypercarbia	12 (8.6%)	03 (2.1%)	0.030
Post-op Nausea Vomiting	35 (25.0%)	47 (33.6%)	0.148
Post-op Shivering	24 (17.1%)	28 (20.0%)	0.539
Post-op Hypothermia	20 (14.3%)	31 (22.1%)	0.089
Delayed Emergence	13 (9.3%)	08 (5.7%)	0.257
Post-op Dizziness	17 (12.1%)	15 (10.7%)	0.707
HDU requirement due to anesthesia	05 (3.6%)	05 (3.6%)	1.000

## DISCUSSION

The study results demonstrated that low flow administration reduced isoflurane consumption and lowered postoperative inflammatory burden while maintaining comparable intraoperative stability. Although the demographic characteristics were similar between both groups a notable difference was observed in postoperative NLR values which were higher in the high flow group. This indicates a stronger inflammatory response when higher fresh gas flows are used.

Bingöl Tanrıverdi et al. (2021) compared low-flow and normal-flow desflurane anesthesia in patients undergoing laparoscopic cholecystectomy and demonstrated that low-flow technique was associated with a significantly smaller increase in postoperative neutrophil-to-lymphocyte ratio (NLR) and platelet-to-lymphocyte ratio (PLR).^9^ In our study, which used isoflurane for maintenance, we observed an analogous pattern: the 24-hour postoperative NLR was significantly lower in the low-flow group compared with the high-flow group. This correspondence suggests that reduced fresh gas flow may attenuate perioperative systemic inflammatory response, regardless of the specific volatile agent used.

Prasad et al. (2020) conducted a randomized, double-blind trial evaluating lung function after low-flow versus high-flow anesthesia in elective surgeries.^8^ Their results indicated that postoperative pulmonary function remained comparable between groups, suggesting that low-flow anesthesia does not compromise postoperative respiratory outcomes. This aligns with our findings: despite using low-flow isoflurane, we did not observe increased rates of postoperative pulmonary complications; intraoperative hypoxia requiring protocol deviation did not occur in any patient. This concordance supports the safety of low-flow techniques from a pulmonary function standpoint.

Doger et al. (2016) assessed the effects of low-flow sevoflurane anesthesia on pulmonary function in patients undergoing laparoscopic abdominal surgery and found no significant differences in pulmonary function tests 2, 8, and 24 hours postoperatively between low-flow and high-flow groups.^19^ Their demonstration that low-flow preserves postoperative lung mechanics supports our observation of stable postoperative respiratory function as no patient was admitted to ICU or HDU due to respiratory issues.

Yoldas et al. (2019) compared hemogram parameters including NLR, PLR and red cell distribution width before and after surgery in low-flow versus high-flow desflurane anesthesia and found that low-flow was associated with significantly lesser inflammatory response as reflected by lower rises in NLR and PLR.^20^ This provides additional independent confirmation of the anti-inflammatory advantage of low-flow anesthesia. Our findings echo this trend, reinforcing the concept that flow rate modulates perioperative inflammatory activation.

Kaşıkara et al. (2022) studied oxidative stress and metabolic damage under low-flow sevoflurane anesthesia and reported that low-flow significantly reduced markers of oxidative stress postoperatively without hemodynamic compromise.^11^ This supports the hypothesis that low-flow reduces oxidative insult and tissue stress, a plausible mechanism for the lower inflammatory markers observed in low-flow groups. In our context, the lower postoperative NLR in low-flow isoflurane cases may reflect similar reductions in oxidative and inflammatory burden.

Ayanoğlu Tas et al. (2021) compared minimal-flow sevoflurane versus desflurane anesthesia in spinal instrumentation surgery and found that minimal-flow anesthesia was safe, cost-effective, and maintained stable hemodynamics and respiratory parameters.^21^ Although their focus was on spinal surgery rather than laparoscopic procedures, their demonstration of low-flow safety across outcomes buttresses our findings of stable intraoperative hemodynamics and acceptable anesthesia adverse effect profiles with low-flow isoflurane.

Kutlusoy S et al. (2022) reported on the reliability of low-flow anesthesia in patients undergoing laparoscopic cholecystectomy under regional practice conditions, noting roughly 50% lower anesthetic gas consumption without increased perioperative risk.^13^ This relates strongly with our results, where low-flow isoflurane consumption was significantly lower than in high-flow, pointing toward resource efficiency and environmental advantage in addition to clinical safety.

Kesimal et al. (2022) evaluated minimal, low, and high-flow anesthesia during elective nasal surgery and observed that tissue oxygen saturation was higher in the minimal-flow group, and patients achieved higher Aldrete scores in the recovery unit.^22^ Although not laparoscopic surgery, this suggests that low-flow may provide better tissue oxygenation and more rapid early recovery. This is consistent with our slightly faster extubation times and stable recovery profiles in the low-flow group.

Gultekin et al. (2021) compared fresh gas flow rates’ effects on airway and cuff pressures in prone-position surgery and found that low-flow or medium-flow techniques did not increase airway complications relative to high-flow.^23^ This supports the safety of low-flow in varied surgical contexts and is consistent with our low incidence of airway-related or respiratory adverse events under low-flow isoflurane.

Upadya et al. (2018) reviewed modern low-flow anesthesia techniques and concluded that with current circle-systems and advanced monitoring low-flow anesthesia is safe, cost-effective, and environmentally friendly.^24^ Their perspective aligns with our findings of reduced volatile use, preserved hemodynamics, lower inflammatory response, and acceptable recovery outcomes. This convergence supports broader adoption of low-flow anesthesia especially in resource-limited settings.

Ability to decrease ICP during laparoscopic procedures;^25^ makes low flow anesthesia an interesting choice for the patients who suffer with intracranial hypertension though our study did not cover that aspect.

In summary, the findings of the present study align strongly with contemporary evidence supporting low flow anaesthesia as a cost-effective technique that decreases the consumption of volatile agents and fresh gas flows while allowing early extubation and reducing inflammation. These findings add to the growing international body of knowledge that supports adoption of low flow strategies particularly in laparoscopic surgery where physiologic changes within the pneumoperitoneum can magnify the differences between flow methods.

### Limitations

First, it was conducted at a single center, which may limit generalizability to different surgical populations, institutional protocols, or equipment capabilities. Second, although the sample size was adequate for detecting differences in inflammatory response and anesthetic consumption, it may not have been powered to detect rare adverse events. Third, postoperative inflammatory markers were restricted to neutrophil-to-lymphocyte ratio, and more detailed immunologic or oxidative stress biomarkers were not assessed, which may have provided deeper insight into the mechanisms underlying reduced inflammation in the low-flow group. More so, NLR is also increased with perioperative stimulus and surgery (though less with laparoscopic procedures as in our study). Fourth, follow-up was limited to early postoperative outcomes; long-term clinical endpoints such as infection rates, recovery quality, or environmental impact were not evaluated. Fifth, it still leaves a question mark about tolerance of low flow anesthesia in patients with significant lung disease. Finally, although confounders were minimized by selecting a single standardized surgical procedure, residual physiological and anesthetic variations cannot be completely excluded.

## CONCLUSION

This study demonstrates that low-flow isoflurane anesthesia in patients undergoing laparoscopic cholecystectomy provides a safe and effective alternative to high-flow techniques. Low-flow anesthesia significantly reduced total isoflurane consumption, time to extubation was reduced and was associated with a lower postoperative inflammatory response. Collectively, these findings support the broader adoption of low-flow anesthesia as a cost-effective, environmentally conscious, and clinically safe practice when appropriate monitoring and modern circle systems are used.

### Author Contributions:

**AH**: Conceptualization, Data Collection, Data analysis, Manuscript writing

**SAJ**: Supervision, conceptualization, Data collection

**AN** Supervision, Statistical analysis, critical review.

**MH**: Literature review, Manuscript writing

All authors have read and approved the final version of the manuscript. They are also accountable for the integrity of the study.
